# Effect of advance care planning video on do-not-hospitalize orders for nursing home residents with advanced illness

**DOI:** 10.1186/s12877-022-02970-3

**Published:** 2022-04-08

**Authors:** Ellen M. McCreedy, Xiaofei Yang, Susan L. Mitchell, Roee Gutman, Joan Teno, Lacey Loomer, Patience Moyo, Angelo Volandes, Pedro L. Gozalo, Emmanuelle Belanger, Jessica Ogarek, Vincent Mor

**Affiliations:** 1 Center for Gerontology and Healthcare Research, Brown University School of Public Health, 121 South Main St, Providence, RI 02912 USA; 2 Department of Health Services, Policy, and Practice, Brown University School of Public Health, 121 South Main St, Providence, RI 02912 USA; 3 Hinda and Arthur Marcus Institute for Aging Research, Hebrew SeniorLife, 1200 Centre St, Boston, MA 02131 USA; 4 Department of Medicine, Beth Israel Deaconess Medical Center, 330 Brookline Avenue, Boston, MA 02215 USA; 5 Department of Biostatistics, Brown University School of Public Health, 121 South Main St, Providence, RI 02912 USA; 6 Oregon Health Sciences University School of Medicine, 3181 SW Sam Jackson Park Rd, Portland, OR 97239 USA; 7 Department of Economics and Health Care Management, Labovitz School of Business and Economics, University of Minnesota Duluth, 1518 Kirby Dr, Duluth, MN 55806 USA; 8 Section of General Medicine, Massachusetts General Hospital, 55 Fruit St, Boston, MA 02114 USA; 9 Harvard Medical School, 25 Shattuck St, Boston, MA 02115 USA

**Keywords:** Pragmatic trial, Nursing home, Dementia, Advance care planning

## Abstract

**Background:**

The purpose of the study is to evaluate the effect of an Advance Care Planning (ACP) Video Program on documented Do-Not-Hospitalize (DNH) orders among nursing home (NH) residents with advanced illness.

**Methods:**

Secondary analysis on a subset of NHs enrolled in a cluster-randomized controlled trial (41 NHs in treatment arm implemented the ACP Video Program: 69 NHs in control arm employed usual ACP practices). Participants included long (> 100 days) and short (≤ 100 days) stay residents with advanced illness (advanced dementia or cardiopulmonary disease (chronic obstructive pulmonary disease or congestive heart failure)) in NHs from March 1, 2016 to May 31, 2018 without a documented Do-Not-Hospitalize (DNH) order at baseline. Logistic regression with covariate adjustments was used to estimate the impact of the resident being in a treatment versus control NH on: the proportion of residents with new DNH orders during follow-up; and the proportion of residents with any hospitalization during follow-up. Clustering at the facility-level was addressed using hierarchical models.

**Results:**

The cohort included 6,117 residents with advanced illness (mean age (SD) = 82.8 (8.4) years, 65% female). Among long-stay residents (*n* = 3,902), 9.3% (SE, 2.2; 95% CI 5.0–13.6) and 4.2% (SE, 1.1; 95% CI 2.1–6.3) acquired a new DNH order in the treatment and control arms, respectively (average marginal effect, (AME) 5.0; SE, 2.4; 95% CI, 0.3–9.8). Among short-stay residents with advanced illness (*n* = 2,215), 8.0% (SE, 1.6; 95% CI 4.6–11.3) and 3.5% (SE 1.0; 95% CI 1.5–5.5) acquired a new DNH order in the treatment and control arms, respectively (AME 4.4; SE, 2.0; 95% CI, 0.5–8.3). Proportion of residents with any hospitalizations did not differ between arms in either cohort.

**Conclusions:**

Compared to usual care, an ACP Video Program intervention increased documented DNH orders among NH residents with advanced disease but did not significantly reduce hospitalizations.

**Trial registration.:**

ClinicalTrials.gov Identifier: NCT02612688.

**Supplementary Information:**

The online version contains supplementary material available at 10.1186/s12877-022-02970-3.

## Background

Advance care planning (ACP) involves ongoing discussions between a patient, a healthcare proxy, and a clinician about the type of care the patient would like to receive if s/he becomes unable to communicate or competently participate in care decision-making [1]. Goals of the ACP process include clarification of the patient’s values and beliefs, designation of a healthcare proxy, and documentation of care preferences [2, 3]. Care preferences may be documented in the form of the living will, a more general statement of wishes to guide a proxy [4], or as medical orders which specify preferences for life‐sustaining treatments [5]. In a nursing home (NH) setting, do-not-hospitalize (DNH) orders are associated with less use of aggressive treatments with little clinical benefit for residents with advanced disease, including terminal hospitalizations [6, 7] and feeding tubes in dementia [8, 9], as well as increased satisfaction with care [10] and quality of life [11].

Unfortunately, there are many barriers to effective ACP. One common barrier is that people are not able to envision health states and treatments which they have not yet experienced [12–14]. The visual images depicted in video ACP support tools may help people better understand the treatment decisions they are being asked to make and the likely outcomes of those decisions [15, 16]. Video support tools have been shown to be efficacious in increasing certainty and stability of advance directive decisions among people with advanced illness when administered by researchers in outpatient and nursing home settings [17–21]. However, little is known about the effectiveness of video support tools as integrated into usual ACP practices in complex NH systems.

The PRagmatic trial Of Video Education in Nursing homes (PROVEN) was an embedded, pragmatic, randomized controlled trial (ePCT) which tested the effectiveness of an ACP Video Program. The primary study outcome was the hospital transfer rates among long-stay residents with advanced illness [22]. While advance directive documentation outcomes was a pre-specified outcome of the PROVEN trial, ultimately only a subset of the trial’s participating NHs (*N* = 110/360) documented these directives in the electronic health record (EHR). Thus, the objective of this report was to compare the acquisition of new DNH orders among all residents with advanced illness in this subset of PROVEN NHs randomized to the ACP Video Program versus usual ACP practices. We also compared the proportion of residents with any hospitalizations during follow-up between arms.

## Methods

### Sample

The PROVEN trial methodology and main results have been described elsewhere [22, 23]. PROVEN used a parallel design. NHs were stratified by corporation, balanced on the distribution of the primary study outcome, and randomized into treatment and control groups in a 1:2 ratio. In this report, we restrict our analyses to 110 of 360 PROVEN facilities owned by a single corporation which consistently used their EHRs to document medical orders for advance directives. Among these NHs, 41 and 69 were randomized to the treatment and control arms, respectively.

### Intervention

Each treatment facility was provided two tablets with five pre-loaded 6–8 min long videos that addressed common decisions in the NH setting: 1. General Goals of Care, 2. Goals of Care for Advanced Dementia, 3. Hospice, 4. Hospitalization, and 5. ACP for Healthy Patients [19]. The PROVEN protocol instructed ACP Champions, predominately social workers, to offer the video at admission, after a hospitalization, every six months, and when decisions were being discussed for which there was a specific video (e.g. hospice) [23]. Efforts to train staff and monitor intervention fidelity have been described elsewhere [22, 24]. Briefly, nursing home champions were trained jointly by the PROVEN implementation team and corporate representatives via webinar, and printed toolkits and pocket reference guides were distributed to champions and other clinical staff to reinforce the training. Champions were instructed to offer the video to residents or proxies within 7 days of admission (short-stay) or every 6 months (long-stay). Other indications for offering the video included when specific treatment decisions arose (hospitalizations, hospice) or under special circumstances (family member visiting). Only 21.9% of residents with advanced illness viewed the video during their followup [22]. Control NHs continued their usual ACP procedures. A waiver of individual informed consent was obtained to conduct this pragmatic trial.

### Population

While all short-stay (≤ 100 days) and long-stay (> 100 days) residents cared for in the facilities between March 1, 2016 and May 31, 2018 were enrolled in the PROVEN, the analyses for the main trial outcomes and this report focused only on residents with advanced disease [22]. Advanced disease patients were defined as having advanced dementia or cardiopulmonary disease (chronic obstructive pulmonary disease (COPD) or congestive heart failure (CFS)) based on routinely collected Minimum Data Set (MDS 3.0) assessments which are ascertained quarterly [25, 26]. Advanced cardiopulmonary disease was defined as having COPD or congestive heart failure with shortness of breath while sitting or supine and needing extensive or total assistance with dressing, transferring, walking or locomotion [27]. Advanced dementia was defined as having either an Alzheimer’s or other dementia diagnosis with advanced cognitive impairment, defined as a score of 3 or 4 on the Cognitive Function Scale [28], and requiring extensive or total assistance with transferring and eating. The date of the first MDS assessment on which a resident met these definitions during the trial implementation period was considered their baseline.

Residents were also excluded if they: had a DNH order at baseline; did not have order data available; were under the age of 65; or were unable to be linked to Medicare enrollment data. The Medicare population includes Fee-for-Service and Medicare Advantage beneficiaries.

### Data sources and variables

Baseline patient characteristics were derived from admission MDS assessments for short-stay residents [29], and from the MDS assessment in which the resident met the criteria for the long-stay cohort [22]. Demographic characteristics included age, gender, and race / ethnicity (White, Black, Asian, Hispanic, other / more than one race, or unknown race). Functional status was assessed using the MDS Activities of Daily Living Scale (range, 0–28, where 28 indicates total functional dependence and 0 indicates no functional dependence) [30]. Mortality risk was assessed using the MDS Mortality Risk Score (range, 0–39, where higher scores indicate higher risk of death) [31].

The main outcome for this report was the acquisition of a new DNH order in the EHR during the follow-up period among residents with advanced illness. The regular expressions used to categorize physician DNH orders within the EHR are listed in Appendix A. The secondary outcome is any hospitalization during follow-up among residents with advanced illness. We used the Medicare Provider Analysis and Review (MedPAR) files to identify hospital admissions for patients in traditional Medicare and those in Medicare Advantage, which account for 92% of Medicare discharges [32, 33]. DNH and hospitalization outcomes were assessed separately for short- and long-stay residents. Short-stay residents were followed up to 100 days from their date of entry into the NH; long-stay residents (> 100 days in nursing home) were followed for up to 12 months.

### Analyses

All analyses were done at the resident-level. Resident characteristics were described using frequencies for categorical variables and means with and standard deviations (SDs) for continuous variables. Logistic regression with covariate adjustments was used to estimate the impact of the resident being a treatment versus control NH on two outcomes: DNH order acquisition and any hospitalization during follow-up. Clustering at the facility-level was addressed using hierarchical models. Analyses for the long-stay and short-stay residents were done separately. To compare outcomes between arms, estimated probabilities and average marginal effects (AME) were reported with standard errors (SEs) and 95% confidence intervals (CIs). Sensitivity analyses were used to demonstrate the robustness of findings under different specifications of the sample.

## Results

### Facility and resident characteristics

**Fig. 1:**
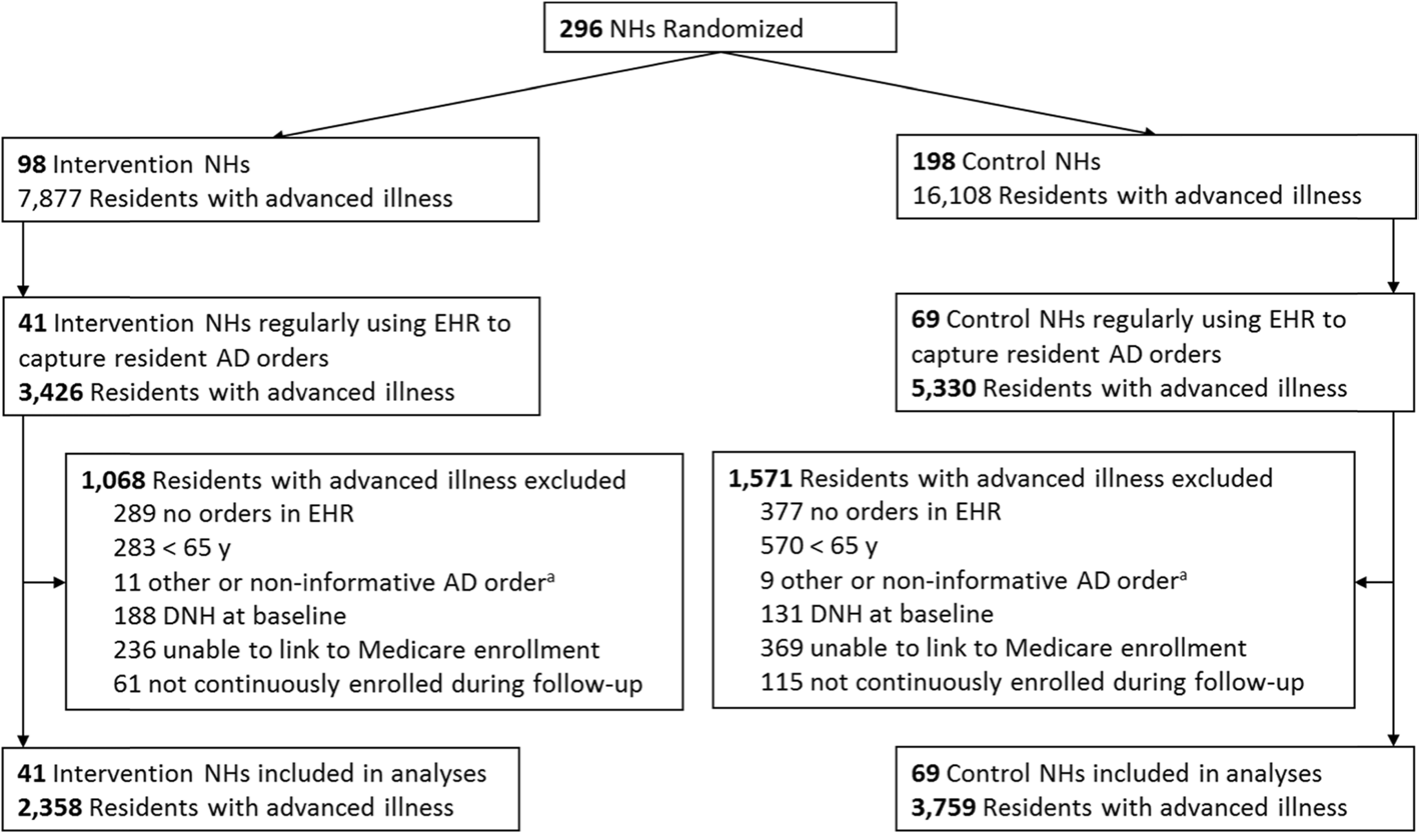
CONSORT diagram of nursing homes and residents. Abbreviations: NH, nursing home; AD, advance directive; EHR, electronic health record. a. Includes 11 treatment and 9 control residents with non-informative orders, referring to an external document without providing the content (e.g., “see POLST”)

Among the 8,756 residents with advanced illness in the 110 eligible NHs (41 treatment facilities, 69 control facilities), we excluded 686 (8%) residents who did not have any EHR data, 853 (10%) who were under the age of 65, 319 (4%) who had a DNH order at baseline, and 781 (9%) who were unable to be linked to Medicare enrollment data or were not continuously enrolled, leaving 6,117 residents for the current analyses (Fig. 1): 3,902 long-stay residents (*N* = 1,485 treatment, *N* = 2,417 control); and 2,215 short-stay residents with advanced illness (*N* = 873 treatment, *N* = 1,342 control). The average age of long-stay residents with advanced illness was 84.0 years (SD, 8.4) in the treatment arm and 83.0 (SD, 8.4) in the control arm (short-stay: 82.0; SD, 8.4 vs. 81.8; SD, 8.0) (Table 1). The majority of residents with advanced illness were White; 92.1% of long-stay residents in the treatment arm, 86.8% of long-stay residents in the control arm; 93.8% of short-stay residents in the treatment arm, 87.4% of short-stay residents in the control arm (Table 1). Our multilevel regression models adjusted for age and race at the resident-level.

**Table 1:** Characteristics of short- and long-stay nursing home residents with advanced illness

	Residents, No. (%)			
	Long-stay^a^ with advanced illness^b^		Short-stay^a^ with advanced illness^b^	
Baseline Characteristics	Intervention (*n* = 1485)	Control (*n* = 2417)	Intervention (*n* = 873)	Control (*n* = 1342)
Age, mean(SD), y	84.0 (8.4)	83.0 (8.4)	82.0 (8.4)	81.8 (8.0)
Female sex	1035 (69.7)	1705 (70.5)	467 (53.5)	739 (55.1)
Race / Ethnicity				
White	1367 (92.1)	2097 (86.8)	819 (93.8)	1173 (87.4)
Black	88 (5.9)	205 (8.5)	31 (3.6)	109 (8.1)
Hispanic	20 (1.3)	77 (3.2)	5 (0.6)	40 (3.0)
Asian	2 (0.1)	7 (0.3)	5 (0.6)	3 (0.2)
Other^c^	6 (0.4)	27 (1.1)	6 (0.7)	10 (0.7)
Unknown	2 (0.1)	4 (0.2)	7 (0.8)	7 (0.5)
Advanced dementia	963 (64.8)	1630 (67.4)	368 (42.2)	660 (49.2)
Advanced CHF or COPD^b^	572 (38.5)	857 (35.5)	522 (59.8)	704 (52.5)
ADL score, mean(SD)^d^	21.6 (3.9)	21.5 (4.0)	20.6 (3.5)	21.0 (3.5)
MRS3 score, mean(SD)^e^	7.7 (2.8)	7.5 (2.7)	7.8 (2.6)	7.6 (2.7)
Days of follow-up, mean(SD)	286.3(124.8)	283.2(126.3)	84.8(30.2)	87.0(28.4)

*Abbreviations*: *ADL* Activities of daily living, *CHF* Congestive heart failure, *COPD* Chronic obstructive lung disease, *MRS3* MDS 3.0 Mortality Risk Score, *NA* Not applicable

^a^Long-stay: greater than 100 days in nursing home; short-stay: 100 or fewer days in nursing home

^b^Advanced illness includes residents with advanced dementia or advanced CHF / COPD

^c^Other includes Native Hawaiian or other Pacific Islander, Native American or Alaska Native, or more than 1 race/ethnicity

^d^The ADL score (0–28) is the sum of scores in 7 domains of function including: bed mobility, dressing, toileting, transfer, eating, grooming, and locomotion. Each is scored on a 5-point scale (0, independent; 1, supervision; 2, limited assistance; 3, extensive assistance; and 4, total dependence). A score of 28 represents complete functional dependence

^e^Range, 0–39; higher scores indicate higher risk of mortality

### Outcomes

At the end of study follow-up (Table 2), a greater proportion of long-stay nursing home residents with advanced illness had new DNH orders in the treatment group (estimated probability 9.3; SE, 2.2; 95% CI 5.0–13.6) than in the control group (4.2; SE, 1.1; 95% CI 2.1–6.3) (average marginal effect, (AME) 5.0; SE, 2.4; 95% CI, 0.3–9.8). Similar differences were observed among short-stay residents with advanced illness. The estimated probability of having a new DNH order among short-stay residents was 8.0% (SE, 1.7; 95% CI 4.6–11.3) in treatment NHs compared to 3.5% (SE 1.0; 95% CI 1.5–5.5) in control NHs (AME 4.4; SE, 2.0; 95% CI, 0.5–8.3). Results were robust to alternative specifications of the sample, including not dropping residents without advance directive orders and removing the age restriction (Appendix Table 2).

**Table 2:** Proportion of residents with new do-not-hospitalize orders and any hospital transfers during follow-up^a^ among residents with advanced illness, by treatment status

	Long-stay residents^b^ with advanced illness^c^			Short-stay residents^b^ with advanced illness^c^		
	% (SE) [95% CI]		AME (SE) [95% CI]	% (SE) [95% CI]		AME (SE) [95% CI]
	Intervention (*n* = 1485)	Control (*n* = 2417)		Intervention (*n* = 873)	Control (*n* = 1342)	
Do-not-hospitalize (DNH) order	9.3 (2.2) [5.0, 13.6]	4.2 (1.1) [2.1, 6.3]	5.0 (2.4) [0.3, 9.8]	8.0 (1.7) [4.6, 11.3]	3.5 (1.0) [1.5, 5.5]	4.4 (2.0) [0.5, 8.3]
Any hospitalization	28.4 (1.6) [25.3, 31.5]	28.8 (1.5) [25.8, 31.7]	-0.4 (2.2) [-4.7, 3.9]	39.9 (1.9) [36.1, 43.6]	38.3 (1.4) [35.6, 41.0]	1.5 (2.4) [-3.1, 6.2]

Reflect estimated probabilities from logistic regression, controlling for resident age and race / ethnicity, with a random effect for nursing homes

*Abbreviations*: *AME* Average marginal effect, *DNH* Do not hospitalize

^a^12-month follow-up for long-stay residents; 100 days follow-up for short-stay residents

^b^Long-stay: over 100 days in nursing home; Short-stay: 100 or fewer days in nursing home

^c^Advanced illness includes residents with advanced dementia or advanced CHF / COPD

The estimated probability of having least one hospitalization did not differ significantly between trial arms in either the long- (28.4% treatment and 28.8% control, AME -0.4; SE, 2.2; 95% CI, -4.7–3.9) and short-stay cohorts (39.9% treatment and 38.3% control, AME 1.5; SE, 2.4; 95% CI, -3.1–6.2). Only 4% (15/363) of residents had any hospitalization after establishing a DNH order.

## Discussion

Residents with advanced illness in NHs randomized to receive an ACP Video Program were more likely to have a new DNH order written during study follow-up compared to residents in NHs randomized to usual care. The intervention did not have a significant effect on hospitalizations.

These findings stand in contrast to what was found in the Educational Video to Improve Nursing home Care in End-stage dementia (EVINCE) trial, a cluster randomized trial of the same ACP Video Program which enrolled 402 residents with advanced dementia and their proxies from 64 Boston area NHs [34]. The EVINCE trial found no effect of the intervention on documented DNH directives after six months of follow-up. However, approximately 50% of residents with advanced dementia in EVINCE had a DNH order documented at baseline. In the current study, only 4% of residents with advance illness had a DNH or comfort care order at baseline, which is consistent with estimates from other nursing home populations [35, 36]. The differences in the EVINCE and PROVEN findings may be partially due to the high baseline use of these orders in the EVINCE study population.

Consistent with the primary results for the PROVEN trial, we did not find an effect of the intervention on the proportion residents with advanced illness who had any hospital transfers [22, 29]. In this subset of NHs that regularly used their EHR to capture advance directive orders, we are possibly underpowered to consider this outcome. However, the potential for disconnect between documentation of DNH and subsequent hospitalizations is an area worth further exploration. Many NHs lack the clinical expertise to safely assess changes in condition and determine whether a resident can be made comfortable without transfer [37]. Telehealth, including palliative care consultations [38], may have the potential to bridge the clinical expertise gap [39–41] but requires more rigorous study. It is also unclear how NH staff interpret DNH and comfort care orders, as a directive to only transfer residents if they cannot be made comfortable in the NH, or as a recommendation to consult with a family member before a resident is transferred [42, 43]. Financial incentives may also encourage hospitalization among residents with DNH orders [44].

Hospitalization rates among newly admitted nursing home residents with DNH orders may be as high as 15% [45]. However, residents with advanced illness with an established DNH order are less likely than similar residents without a DNH order to be hospitalized at the end of life or die in a hospital. A study of end-of-life transitions among nursing home decedents with advance illness, found only 7% of those with a DNH order experience any burdensome transition at the end of life, compared to 19% of all residents in the decedent sample [46]. A site of death analysis of a similarly comorbid population, found only 4.6% of residents with a DNH order died in a hospital compared to 20.5% of residents without a DNH order [35]. In our sample, hospitalizations were similarly rare (4%) among persons with advanced illness once they had a DNH order. DNH preferences are documented late in the dying process [47] and, thus, may not result in a detectable difference in hospitalizations in a study of residents who are not actively dying. A recently published decedent analysis from the PROVEN study revealed residents with advanced illness in NHs randomized to receive the intervention were less likely to be transferred to the hospital in the last 90 days of life compared to similar residents in NHs randomized to the control arm [48]. While there is plenty of room for rigorous debate on the effectiveness of ACP interventions in altering decision-making and the timing advance care planning decisions [49], the association between presence of a DNH order and the quality of death that most people would prefer is well-established.

This analysis uses a subset of the original study facilities, identified post-randomization. Thus, characteristics of residents in treatment and control facilities are not as balanced at baseline as in the full trial sample, particularly on race / ethnicity. Because of this imbalance, all reported estimated probabilities and marginal effects were adjusted for age and race / ethnicity, with a random effect to account for clustering at the facility level. Further potential for selection bias was explored in the sensitivity analyses, which were robust to primary findings. Study power calculations were not conducted based on this sample or outcome. Non-significant findings may be a consequence of small effect size. Consistent with pragmatic trial paradigms, we leveraged existing workflows to implement the intervention which resulted in a social worker offering the video to residents / proxies and leading the discussion of care preferences 87% of the time [50]. Low physician engagement may affect the quality of ACP conversations and communication of resident wishes during a crisis, but this is beyond the scope of the current study. Finally, in our sample we find that within a corporation in which all NHs are using the same EHR, NHs with more racial and ethnic diversity were less likely to be using their EHR to document resident advance directives or code status. This is important for the design of future pragmatic trials which should consider data availability of specific modules within a EHR when focusing on equity of study design [51] and generalizability of findings [52].

## Conclusions

Use of DNH orders among nursing home residents with advanced illness is low. An ACP Video Program may improve documentation of DNH orders in a population of residents with advanced disease. More research is needed to understand the optimal timing for the delivery of the ACP Video Program, and the relationship between documentation of preferences for comfort care or DNH orders in the EHR and subsequent hospitalization decisions. We lack a common measure of code or directive status for all NH residents in the US, which results in reliance on EHR data which may underrepresent more diverse facilities.

## Supplementary Information

**Additional file 1.**

## Data Availability

The datasets are subject to use agreements and can not be made public However, the analytic files are available in the Brown repository.

## Abbreviations

NH: Nursing home;
HER: Electronic health record
ACP: Advance care planning
DNH: Do-not-hospitalize
PROVEN: Pragmatic Trial of Video Education in Nursing Homes
SD: Standard deviation
CI: Confidence interval
SE: Standard error
AME: Average marginal effects
MDS: Minimum Data Set
COPD: Chronic obstructive pulmonary disease
CFS: Congestive heart failure
ePCT: Embedded pragmatic, randomized controlled trial
EVINCE: Educational Video to Improve Nursing home Care in End-stage dementia
