# Supplementary material for: Effect of advance care planning video on do-not-hospitalize orders for nursing home residents with advanced illness
Source: BMC Geriatr. 2022 Apr 8;22:298. doi: 10.1186/s12877-022-02970-3 (PMC8991654; doi:10.1186/s12877-022-02970-3)
Supplement: Supplementary file 1 — Additional file 1. [file 12877_2022_2970_MOESM1_ESM.docx]

**Appendix Table 1.** Regular expressions used to categorize physician do-not-hospitalize orders in electronic health record

| Status | Regular Expressions | Physician orders captured by regular expression |
| --- | --- | --- |
| DNH | DNH  DNT  (NO\|NOT\|not to be)[[:space:]]*(acute\|severe\|comfort)?(transfer\|transport\|hospital\|send to hospital\|send to the hospital\|hsp\|send to ER\|hosp)  decline[^[:punct:]]*hospital  (send\|trans\|hospital\|hosp.\|hospital..\|transfer:)[^[:punct:]]*(acute\|severe\|comfort)[^[:punct:]]*(only)?  (only)?[[:space:]]*(send\|trans\|hospital)[^[:punct:]]*(only)?[^[:punct:]]*(acute\|severe\|comfort)  (acute\|severe\|comfort)[^[:punct:]]*(trans\|hospital)[^[:punct:]]*(only)?  treat[[:space:]]*in[[:space:]]*house | DNH  DNT: DO NOT TRANSFER  DNT  DNTH  Do not hospitalize  no hospitalization  Do not transfer to hospital  not send to ER  Do not transfer  no acute hospital transfer  Do not transfer treat with options available outside of hospital  Do not transport to hospital  no hsp transfer except for acute  do not hospitalize (except for comfort)  do not hosp/ wants abx  Patient declines hospitalization  send to hospital for acute injury only  Transfer for acute injury only  hospital for acute injury only  transfer to hospital for acute care only  transfer for acute injury  transport for acute injury  may transfer to hospital for acute need.  may transfer to hospital for hosp level of care for acute problems  transfer to hospital for evaluation of acute injury and acute reversible condition  hospitalize acute injury  send to hospital for acute care as needed  transfer to hosp. for acute  transfer to hosp. for eval of acute  hospital.. for acute  hospital transfer: acute  transfer to hospital for evaluation of acute injuy olny  transfer to hospital for severe pain or sever symptoms that cannot be controlled here  transfer only for acute injury  only transfer for acute injury  transfer to hospital only if comfort needs can not be met in current location per post  transfer to hospital if comfort measures cannot be met in current location  acute injury transfer only |

**Appendix Table 2.** Proportion of residents with new do-not-hospitalize orders during followup **(**alternative specifications)

|  | Long-stay with advanced illness | | | | Short-stay with advanced illness | | | |
| --- | --- | --- | --- | --- | --- | --- | --- | --- |
|  | n | Intervention  % (SE) [95% CI] | Control  % (SE) [95% CI] | AME (SE)  [95% CI] | n | Intervention  % (SE) [95% CI] | Control  % (SE) [95% CI] | AME (SE)  [95% CI] |
| Primary Specification* | 3902 | 9.3 (2.2)  [5.0, 13.6] | 4.2 (1.1)  [2.1, 6.3] | 5.0 (2.4)  [0.3, 9.8] | 2215 | 8.0 (1.7)  [4.6, 11.3] | 3.5 (1.0)  [1.5, 5.5] | 4.4 (2.0)  [0.5, 8.3] |
| Assign full code if missing order† | 4197 | 8.5 (2.0)  [4.5, 12.5] | 3.9 (1.0)  [2.0, 5.9] | 4.6 (2.3)  [0.1, 9.0] | 2453 | 7.2 (1.6)  [4.1, 10.3] | 3.2 (0.9)  [1.4, 5.0] | 4.0 (1.8)  [0.4, 7.6] |
| No age restriction‡ | 4223 | 9.1 (2.1)  [4.9, 13.3] | 4.1 (1.0)  [2.1, 6.1] | 5.0 (2.4)  [0.4, 9.7] | 2387 | 7.4 (1.6)  [4.3, 10.5] | 3.5 (1.0)  [1.5, 5.4] | 3.9 (1.9)  [0.3, 7.6] |
| Drop two control NHs with majority African American residents§ | 3835 | 9.2 (2.2)  [4.9, 13.5] | 4.4 (1.1)  [2.2, 6.5] | 4.9 (2.4)  [0.1, 9.6] | 2163 | 7.9 (1.7)  [4.6, 11.3] | 3.7 (1.0)  [1.6, 5.7] | 4.3 (2.0)  [0.3, 8.2] |

Reflect estimated probabilities from logistic regression, controlling for resident age and race / ethnicity, with a random effect for nursing homes

*Primary specification includes nursing homes in which at least 75% of study participants had an advance directive order. Within the sub-sample of eligible nursing homes, if residents did not have an order before their end of study follow-up they were dropped from the analysis. Residents were also dropped from the analysis if <65 years old.

†Same as primary specification except residents without medical orders are assigned full code status (not DNH or comfort care) at end of followup.

‡Same as primary specification except no age restriction (includes residents under the age of 65)

§Same as primary specification except remove two nursing homes with majority African American residents from control group (no nursing homes with majority African American residents in treatment group)
